# Decoding the Multiple Identities and Crosstalk of Organokines in Obesity-Related Type 2 Diabetes Mellitus

**DOI:** 10.14336/AD.2024.1138

**Published:** 2026-01-05

**Authors:** Yu-Qing Ni, Jun-Kun Zhan, You-Shuo Liu

**Affiliations:** ^1^Department of Geriatrics, The Second Xiangya Hospital of Central South University, Changsha, Hunan, China.; ^2^Institute of Aging and Age-related Disease Research, Central South University, Changsha, Hunan, China.

**Keywords:** organokines, adipokines, myokines, hepatokines, obesity, T2DM

## Abstract

Obesity causes an imbalance in the expression and secretion of several organokines, which in turn contributes to the development of metabolic disorders such as type 2 diabetes mellitus. Organokines are produced by corresponding organs and affect systemic metabolic homeostasis. Diverse organokines play a crucial role in the communication between adipose tissue, skeletal muscle and other organs. In this review, we discuss the biological properties of specific organokines such as adipokines, hepatokines, and myokines. We also highlight the cumulative roles and crosstalk of organokines in obesity-related T2DM. Moreover, we attempt to identify the diagnostic and therapeutic potential of obesity-related T2DM from the perspective of organokines.

## 1. Introduction and Background

Obesity is featured by excessive adipose tissue and is a complex process involving both intrinsic genetic and external environmental factors. It is closely related with chronic complications, such as dyslipidemia, hyperglycemia, and hypertension [1]. According to the Global Burden of Disease Obesity Collaborators, more than 603.7 million adults were obese and about 4 million deaths were associated with an increase in body mass index (BMI) [2]. Multiple studies have demonstrated that obesity contributes to the occurrence and progression of type 2 diabetes mellitus (T2DM) [3]. We are in the midst of a global obesity epidemic, with an increased risk of developing related metabolic diseases. Dysregulation of glucose and lipid metabolism are two core factors of metabolic diseases. The disability and mortality rates caused by these diseases impose a serious economic burden on individuals, families and societies. Therefore, considerable research efforts are needed to understand the pathogenesis and role of obesity and related diseases.

Organokines are exclusively or primarily produced by their respective organs and influence systemic metabolic homeostasis through endocrine, paracrine, and autocrine manners. For example, adipose tissue releases a range of biologically active substances, known as adipokines. Peptides and cytokines produced by skeletal muscle are called as myokines. Moreover, liver-derived bioactive proteins are known as hepatokines. Organokines are novel modulators involved in the organ interaction and play an important role in tissue metabolic homeostasis [4]. In a high-glucose and high-fat environment, cells exert a variety of effects on the whole body through oxidative stress, endoplasmic reticulum stress, and other pathological processes. Organokines can be divided into pro-inflammatory and anti-inflammatory factors based on their roles in regulating inflammation. However, it is worth noting that some organokines, such as fetuin-A and interleukin-6 (IL-6), exert both pro-inflammatory and anti-inflammatory roles depending on the type of receptor or tissue [5, 6]. In this review, we discuss the biological properties of specific organokines such as adipokines, hepatokines, and myokines. We also focus on the cumulative roles and crosstalk of organokines in obesity-related T2DM. Moreover, we attempt to identify the diagnostic and therapeutic potential of metabolic diseases from the perspective of organokines.

## 2. Definition and Roles of organokines

Obesity is accompanied by alterations in organokines, such as adipokines, hepatokines, and myokines. Each organokine has a special molecular structure and plays a different physiological function. Moreover, these organokines can interact with each other to form a complex cross regulation, thereby maintaining system homeostasis. Here, we summarize and discuss the characteristics of several important organokines (Table 1), which are closely involved in obesity.

**Table 1:** The characteristics of organokines.

Organokines		Primary source	Binding partner or receptor	Mechanism of actions	Function	Ref.
Adipokines	Leptin	Adipocytes	Leptin receptors	Regulates the hypothalamic-sympathetic nervous system axis and activates AMPK in skeletal muscle cells	Inhibits appetite and regulates fatty decomposition and synthesis	[16]
	**Adiponectin**	Adipocytes	AdipoR1 and AdipoR2	Phosphorylates p38 MAPK and AMPK, and raises activity of PPAR-α ligand	Influences glycolipid metabolism, energy balance, and cardiometabolic function	[24, 25]
	**Resistin**	Monocytes and macrophages (human), adipocytes (rodent)	Unknown	Regulates the suppressor of SOCS3 and affects the MAPK, PPAR-γ, PI3K signaling pathways.	Modulates endothelial dysfunction, obesity-related insulin resistance, and CVD	[33, 34]
	**CTRPs**	Adipocytes	Unknown	Modulates AMPK-mediated autophagy induction, activates PI3K-protein kinase B signaling pathway, etc.	Regulates glycolipid metabolism, inflammation, and cardiovascular function	[38, 42, 43]
Hepatokines	SHBG	Liver	Megalin and G-protein coupled receptors	Be associated with hepatic insulin sensitivity	Regulates obesity, osteoporosis, and metabolic syndrome	[45]
	**SEPP**	Liver	Selenium	Exerts vital roles in redox homeostasis via phospholipid peroxidase activity	Interferes with glucose metabolism	[49, 51, 52]
Myokines	Irisin	Muscle	FNDC5	Up-regulates the expression of mitochondrial UCP1	Increases energy expenditure, improves insulin resistance and glucose tolerance	[56, 57]
	**Myostatin**	Muscle	Unknown	Negatively mediates Akt pathway, which enhances the activity of the ubiquitin-proteasome system and induces protein synthesis	Regulates glucolipid metabolism	[59]
	**BDNF**	Muscle	Trk B	Activates AMPK signaling pathway by promoting phosphorylation of AMPK and its downstream signaling molecule acetyl-CoA carboxylase β	Increases insulin sensitivity, influences energy metabolism, suppresses appetite	[64, 65]
**Adipo-hepato-myokine**	FGF-21	Adipocytes, Liver, Muscle	FGFRs	Binds to FGFRs with the help of co-receptor β-Klotho	Plays a key role in glucolipid regulation and energy metabolism	[72]
Adipo- myokine	IL-6	Adipocytes, Muscle	IL-6R	Exerts both pro-inflammatory and anti-inflammatory effects	Be closely associated with the occurrence of metabolic disorders	[25, 76]
	**TNF-a**	Adipocytes, Muscle	TNF receptor	Inhibits AMPK, attenuates the insulin-stimulated tyrosine phosphorylation of IRS1	Regulates total glucose metabolism, lipolysis, and lactate production, as well as modulates insulin resistance	[78, 79]
Adipo-hepatokine	Fetuin-A	Adipocytes, Liver	Unknown	Exerts pro-inflammatory or anti-inflammatory roles according to its receptor or target tissue	Acts as a predisposition site for T2DM, obesity, and insulin resistance	[82, 85]
	**Chemerin**	Adipocytes, Liver	ChemR23	Exacerbates glucose intolerance and disturbs insulin signaling	Acts as a link between inflammation, obesity, and other metabolic disorders	[88, 90]
**Hepato-myokine**	Follistatin	Liver, Muscle	Unknown	Promotes hypertrophy of skeletal muscle fibers, inhibits TGF-β/myostatin signaling pathway	Regulates skeletal muscle mass and energy metabolism	[92, 93]

### 2.1 Adipokines

Adipose tissue is not only an energy storage organ, but also a vital endocrine organ. It is divided into white adipose tissue (WAT) and brown adipose tissue (BAT), among which WAT stores excess energy and has endocrine function. With the progression of studies, the endocrine function of adipose tissue has received more and more attention. Adipokines refer to bioactive factors or factor-like molecules produced and secreted by adipose tissue that are involved in energy homeostasis, fat metabolism, insulin sensitivity, and inflammatory regulation [7]. Remarkably, adipose tissue can generate hundreds of adipokines, such as leptin, adiponectin, resistin, adipocyte fatty acid binding protein (A-FABP), tumor necrosis factor α (TNF-α), asprosin, chemerin, retinol binding protein 4 (RBP4), vaspin, zinc-α2-glycoprotein (ZAG), C1q/TNF-related proteins (CTRPs), and *etc*. In physiological state, adipokines can act on tissues and organs such as brain, liver, skeletal muscle, cardiovascular and immune system, endocrine and pancreas. When the body is in a state of decompensation or disease, such as obesity and metabolic syndrome, multiple adipokines secretion function becomes impaired, thereby increasing the disorder of glucose and lipid metabolism [8]. Obesity leads to remarkable changes in the adipokine profile, resulting in decreased levels of anti-inflammatory adipokines and increased levels of pro-inflammatory adipokines. Below, we briefly introduce several adipokines and discuss their metabolic regulatory properties.

### 2.1.1 Leptin

Leptin is a 16 kDa non-glycosylated peptide hormone that is produced mostly in WAT [9], but can also be expressed by BAT [10], mammary glands [11], stomach [12], and skeletal muscle [13]. Circulating leptin concentrations correlate well with absolute adipose tissue mass, suggesting that leptin is an outstanding biomarker of body fat content. Obesity, satiety, glucocorticoids, insulin, and acute infection increase leptin levels, whereas cold stimulation, fasting, and testosterone decrease leptin levels [14]. Leptin receptors consist of six isoforms (LEPR a-f) and are commonly expressed in most tissues of the body [15]. Leptin is known to play an important role in regulating energy expenditure, appetite, and satiety. On the one hand, leptin inhibits appetite through the hypothalamic-sympathetic nervous system axis, reducing food intake and increasing calorie consumption. On the other hand, leptin activate AMP-activated protein kinase (AMPK) in skeletal muscle cells, thereby enhancing fatty acid decomposition and inhibiting fat synthesis [16]. Besides, increasing evidence indicates that leptin exerts a pleiotropic role in neuroendocrine function, fertility, and reproductive function [17, 18]. Fortunately, leptin supplementation has been shown to reverse obesity and improve metabolism in children with congenital leptin deficiency [19]. However, it was reported that the circulating level of leptin was increased in obese individuals, possibly due to leptin resistance or tolerance [17, 20]. Administration of metreleptin, a recombinant methyl-human leptin hormone, only shows a slight benefit with a decrease in glycated hemoglobin in subjects with leptin resistance or hyperleptinemia [21].

### 2.1.2 Adiponectin

Adiponectin, a 30-kDa monomeric glycoprotein, is a classic anti-inflammatory adipokine that is present in high concentrations in plasma [22]. Adiponectin receptors (AdipoR) are homologous receptors of adiponectin, where AdipoR1 is predominantly expressed in skeletal muscle and AdipoR2 is mainly expressed in liver [23]. Upon binding to the receptors, adiponectin triggers a whole train of events, including phosphorylation of p38 mitogen-activated protein kinase (MAPK) and AMPK, and raised activity of peroxisome proliferator-activated receptor alpha (PPAR-α) ligand [24]. Accumulating evidence indicates that adiponectin plays vital roles in glycolipid metabolism, energy balance, and cardiometabolic function [25]. Moreover, adiponectin and leptin have been shown to have a close interaction in obesity pathology and related metabolic complications. Accordingly, there is an increased risk of hyperglycemia, dyslipidemia, and cardiovascular disorders with a decreased adiponectin level [26, 27].

### 2.1.3 Resistin

Steppan *et al.* first demonstrated that resistin was a small circulating protein specifically secreted and expressed by adipocytes in 2001 [28]. The expression patterns of resistin in humans and rodents are very different. Human resistin is primarily produced by monocytes and macrophages infiltrated in adipose tissue, while murine resistin is secreted mainly by adipocytes [29]. A variety of hormones or molecules can affect resistin expression, such as hyperglycemia, insulin, TNF-α, IL-6, estrogen, and lipopolysaccharide [30-32]. Resistin, a pro-inflammatory adipokine, is involved in the activation of suppressor of cytokine signaling 3 (SOCS3) in glucose metabolism [33]. Besides, the regulation also involves MAPK, PPAR-γ, phosphatidylinositide-3-kinase (PI3K) signaling pathways. Resistin is a modulator of endothelial dysfunction, obesity-related insulin resistance, and CVD [34]. Resistin can affect insulin action directly or indirectly by acting on skeletal muscle and adipose tissue. Hyperinsulinemia and hypertriglyceridemia are caused by abnormal lipid metabolism and decreased skeletal muscle insulin sensitivity when resistin is overexpressed. Notably, although resistin levels are significantly elevated in T2DM, the relationship between resistin and insulin resistance, obesity or hyperlipidemia remains unclear.

### 2.1.4 C1q/TNF-related proteins

In 2004, Wong *et al.* first identified a family of adipokines as CTRPs, which consists of 15 members (CTRP1-15) [35]. Similar to TNF and adiponectin, all members share a C1q globular domain [36]. Most CTRPs are generally expressed in murine and human adipose tissue with diverse levels in the circulation according to genetic background, gender, metabolic status, and *etc* [37]. CTRPs are associated with the regulation of glycolipid metabolism, inflammation, and cardiovascular function [38]. Studies have found that CTRP3 expression is enhanced in patients with glucose metabolism disorders and is related with a series of metabolic risk factors [39]. Moreover, the level of circulating CTRP3 in patients with CVD were markedly lower than those control subjects [40]. Increased CTRP6 concentration has been shown to inhibit glucose disposal in mouse peripheral tissues[41]. In addition, Jung *et al*. revealed that CTRP9 alleviated hepatic steatosis by AMPK-mediated autophagy induction to relieve endoplasmic reticulum stress [42]. Overexpression of CTRP12 activated PI3K-protein kinase B signaling pathway, thereby inhibiting gluconeo-genesis and promoting glucose uptake in cultured hepatocytes and adipocytes [43]. Unfortunately, the exact role of some CTRPs in modulating diverse signaling pathways associated with metabolic disorders is still in its infancy.

### 2.2 Hepatokines

Hepatokines, liver-derived proteins, play prominent roles in coordinating systemic metabolic syndrome such as obesity, dyslipidemia, hypertension, and insulin resistance. Cultured hepatocytes secrete more than 500 proteins. However, only few have been studied as hepatokines and only a small fraction are involved in obesity-related metabolism. Among them, fibroblast growth factor-21 (FGF-21), fetuin-A, selenoprotein P (SEPP), sex hormone-binding globulin (SHBG), hepatocyte-derived fibrinogen-related protein 1, leukocyte cell-derived chemotaxin 2 (LECT2), and angiopoietin-related growth factor (AGF), are considered to be the most important hepatokines in modulating the pathogenesis of obesity. In this section, we compile and discuss the identification and functional characterization of several hepatokines to better understand the pathogenesis of obesity-related metabolic diseases.

### 2.2.1 Sex hormone-binding globulin

SHBG is a 90-kDa glycoprotein whose main role is believed to be the transport of sex steroids. SHBG binds to testosterone, estradiol, and other steroids in serum with varying affinity. SHBG can bind to G-protein coupled receptors on the plasma membrane. Megalin, also known as low-density lipoprotein receptor-associated protein 2, is an SHBG receptor. It promotes intracellular endocytosis of SHBG-binding steroids [44]. In addition to steroid transport, emerging evidence indicates that SHBG is involved in regulating a variety of important processes. SHBG serum level is modulated by sex hormone imbalance and metabolic disorders. Besides, it has been reported that SHBG concentration is closely related to hepatic insulin sensitivity [45]. As a mediator between various endocrine tissues, SHBG plays a crucial pathophysiological role in obesity, osteoporosis, metabolic syndrome, and *etc.* [46].

### 2.2.2 Selenoprotein P

Selenoproteins are polypeptides containing at least one selenocysteine residue and exist in organisms ranging from bacteria to mammals [47]. Some selenoproteins exhibit redox-related enzyme activities and play important effects in cellular reactive oxygen species (ROS) scavenging system [48]. SEPP is a unique selenoprotein that contains a large number of selenocysteine residues, which makes SEPP a dominant and efficient vector. It has been reported SEPP exerts vital roles in redox homeostasis due to the phospholipid peroxidase activity [49]. Imbalanced SEPP levels, whether high or low, have been associated with the progression of several metabolic diseases, respectively. It has been confirmed that decreased SEPP level promotes oxidative stress disorders [50]. Moreover, emerging evidence proves that enhanced SEPP expression interferes with glucose metabolism, leading to T2DM [51, 52]. Therefore, a growing number of studies are exploring the potential of SEPP as a predictive or diagnostic biomarker and as a therapeutic target.

### 2.3 Myokines

Skeletal muscle regulates metabolic processes and energy in the human body. It is currently recognized that the organ is able to produce molecules important functional molecules, known as myokines [53]. Myokines can not only act on the skeletal muscle to modulate the metabolism of glucose, lipid and protein, but also reach the periphery through blood circulation and serve as messengers of dialogue between skeletal muscle and adipose tissue, liver, brain, and heart [54]. The classic member of this family is IL-6. Recently, other myokines, such as irisin, myostatin, brain-derived neurotrophic factor (BDNF), have been described. Emerging evidence indicates that myokines are closely associated with the occurrence and development of obesity-related metabolic diseases.

### 2.3.1 Irisin

Irisin is a novel myokine that is produced in large quantities in response to exercise. Exercise induces the expression of fibronectin type III domain containing protein 5 (FNDC5) by promoting PPAR-γ coactivator-1 alpha (PGC-1α), thereby stimulating the secretion of irisin [55]. Irisin up-regulates the expression of mitochondrial uncoupling protein 1 (UCP1), which enhances thermogenesis and energy consumption in the BAT and skeletal muscle [56]. Once released into the circulation, irisin acts on white adipocytes to induce the browning response, resulting in increased energy expenditure, and improved insulin resistance and glucose tolerance. Increasing evidence demonstrates the favorable effects of irisin on cardiovascular disease, metabolic diseases, and NAFLD [57].

### 2.3.2 Myostatin

Myostatin is a member of transforming growth factor beta (TGF-β) superfamily. As a negative regulator of muscle mass, it plays an important role in the growth and development of skeletal muscle [58]. When the level of myostatin is elevated, it leads to increased inhibition of growth and degradation of skeletal muscle, while skeletal muscle mass increases dramatically with a decreased level of myostatin. It exerts enormous effects in integrating anabolic and catabolic reactions. Myostatin negatively mediates Akt pathway, which enhances the activity of the ubiquitin-proteasome system and induces protein synthesis [59]. Recent studies have shown that myostatin is also involved in glucolipid metabolism, leading to obesity, T2DM, and insulin resistance [60, 61].

### 2.3.3 BDNF

BDNF is a vital member of the neurotrophic factor family together with neurotrophin and nerve growth factor [62]. BDNF maintains neuronal survival, regulates neuronal growth and differentiation, and thus mediates memory and learning. Pedersen *et al*. reported that exercise induced the release of other myokines, which in turn crosses the blood-brain barrier to stimulate cerebral BDNF secretion [63]. More recent studies have shown that BDNF also modulates organism metabolism by increasing insulin sensitivity, influencing energy metabolism, suppressing appetite, which exerting a good hypoglycemic effect [64]. In terms of molecular mechanism, BDNF activates AMPK signaling pathway by promoting phosphorylation of AMPK and its downstream signaling molecule acetyl-CoA carboxylase β [65].

### 2.4 Organokines with multiple identities

**Figure 1.**
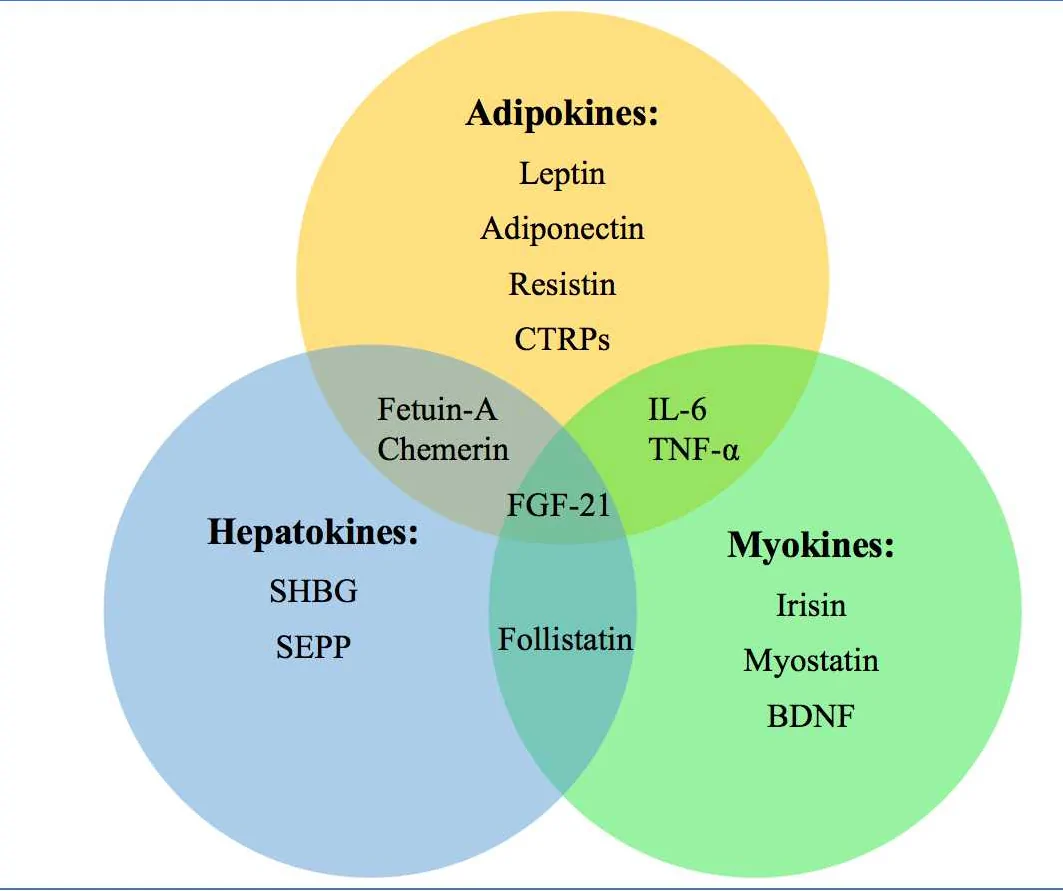
The multiple identities of organokines.

A number of organokines have multiple identities that can be secreted by different organs (Fig. 1). There is considerable overlap between adipokines, hepatokines, and myokines. Some cytokines secreted by adipocytes are also secreted by hepatocytes and skeletal muscle cells. FGF-21 is classic described member of the “adipo-hepato-myokine” family. Besides, IL-6 and TNF-a are classified as adipokines and myokines as they can be produced by adipose and skeletal muscle. Moreover, fetuin A and chemerin are identified as “adipo-hepatokine”, while follistatin is categorized as “hepato-myokine”.

### 2.4.1 Adipo-hepato-myokine

A family of 22 members of FGF has been identified in humans. FGF-21, a protein with 181 amino acid residues, is commonly expressed in the liver and other organs, such as adipose tissue and skeletal muscle [66]. The expression of FGF-21 is mainly regulated by two signaling axis. On the one hand, the increase in the secretion and expression of FGF-21 is mediated by the exchange protein directly activated by the cyclic adenosine monophosphate/protein kinase A pathway through transcriptional mechanisms [67]. On the other hand, activation of PPAR-α pathway can also enhances FGF-21 gene level [68]. Generally, the signaling pathway is induced and activated in a series of alterations, such as fasting, overnutrition, mitochondrial stress, and hyperinsulinemia [69-71]. FGF-21 binds to fibroblast growth factor receptors (FGFRs) with the help of co-receptor β-Klotho, a transmembrane glycoprotein, and thus plays a key role in glucolipid regulation and energy metabolism [72].

### 2.4.2 Adipo-myokine

IL-6 was the first described myokine, which is essential for muscle performance during contraction [73]. It facilitates intramuscular oxidation via activating AMPK in skeletal muscle [74]. Subsequently, IL-6 was shown to be produced by a variety of cells, including adipose cells. IL-6 has both pro-inflammatory and anti-inflammatory effects, making its role ambiguous and contradictory [25]. As an adipokine, IL-6 functions as a pro-inflammatory label and is correlated to obesity, insulin resistance, and T2DM. It is stimulated by the activation of nuclear factor-kappa B (NF-κB) and suppressing the expression of glucose transporter type 4 (GLUT4) and insulin receptor substrate 1 (IRS1) in adipocytes [75]. Myokine IL-6 promotes the secretion of IL-10, an inhibitor of TNF-α, representing an anti-inflammatory role in muscle skeletal. Increasing evidence indicates IL-6 is closely associated with the occurrence of metabolic disorders [76].

TNF-α, an inflammatory cytokine, is produced mainly by visceral fat infiltrated by macrophages [77]. TNF-a activates the MAPK signaling pathway through the mediation by TNF receptor, including extracellular regulated protein kinase 1/2 (ERK1/2) and JNK in adipose tissue. It is corelated with increased total glucose metabolism, enhanced lipolysis, and accelerated lactate production in adipocytes [78]. As a myokine, TNF-a inhibits AMPK activity, thereby decreasing fatty acid oxidation and ACC phosphorylation, and eventually resulting in insulin resistance in skeletal muscle [79]. TNF attenuates the insulin-stimulated tyrosine phosphorylation of IRS1 in adipose tissue and muscle, resulting in the occurrence of insulin resistance.

### 2.4.3 Adipo-hepatokine

Fetuin-A is a 64-kDa glycoprotein produced primarily in liver, which acts as a metabolic modulator through the interaction between the liver and other organs [80]. Subsequent studies have shown that adipose tissue can also secrete fetuin-A [81]. The fetuin gene, located on chromosome 3q27 in humans, is a predisposition site for T2DM, obesity, and insulin resistance [82]. Increasing evidence proves that the level of fetuin-A is closely associated with systemic biology and pathology. The production of fetuin-A is regulated by a variety of molecules and hormones, among which TNF-α, IL-6, and IL-1β can reduce the expression of fetuin-A, while thyroid hormones can increase fetuin-A expression [83, 84]. Importantly, the roles and levels of fetuin-A are quite different in several diseases. It has been demonstrated that fetuin-A exert pro-inflammatory or anti-inflammatory roles according to its receptor or target tissue [85]. For example, fetuin-A plays a pro-inflammatory role in metabolic syndrome and NAFLD. On the contrary, it exhibits an anti-inflammatory effect in dementia and autoimmune disorders [5]. Fetuin-A is also considered as an indicator and biomarker for insulin resistance, cardiovascular and neurodegenerative diseases [86].

Chemerin, encoded by retinoic acid receptor responder 2 (Rarres2), was described as an adipokine with diverse secretory modes [87]. Later, it was also shown to be a hepatokine secreted by hepatocytes. Chemerin is activated by serine proteases and inflammation, exacerbating glucose intolerance and disturbing insulin signaling. The action occurs through chemerin receptor 23 (ChemR23), which is mainly concentrated in adipose tissue and hepatocytes [88]. It has been reported that the level of chemerin is elevated in obesity, insulin resistance, and metabolic syndrome [89]. Increased expression has been shown to be positively associated with deleterious alterations in cytokine, glucose, and lipid, which is also a link between inflammation, obesity, and other metabolic disorders [90].

### 2.4.4 Hepato-myokine

Follistatin, a hepatokine and myokine, is released during physical activity. It is a monomeric glycosylated protein with a high affinity for binding and neutralizing activins [91]. Follistatin promotes hypertrophy of skeletal muscle fibers because of its affinity with myostatin, which neutralizes and enhances skeletal muscle mass [92]. Moreover, follistatin can also induce brown adipose properties and regulate energy metabolism due to the role in inhibiting TGF-β/myostatin signaling pathway [93]. Further molecular analysis identified two possible distinct pathways responsible for browning. On the one hand, follistatin promotes increased phosphorylation of p38 MAPK and ERK1/2 [94]. On the other hand, it regulates the induction of WAT browning and classical BAT via targeting Myf5+ precursor pools [95].

**Figure 2.**
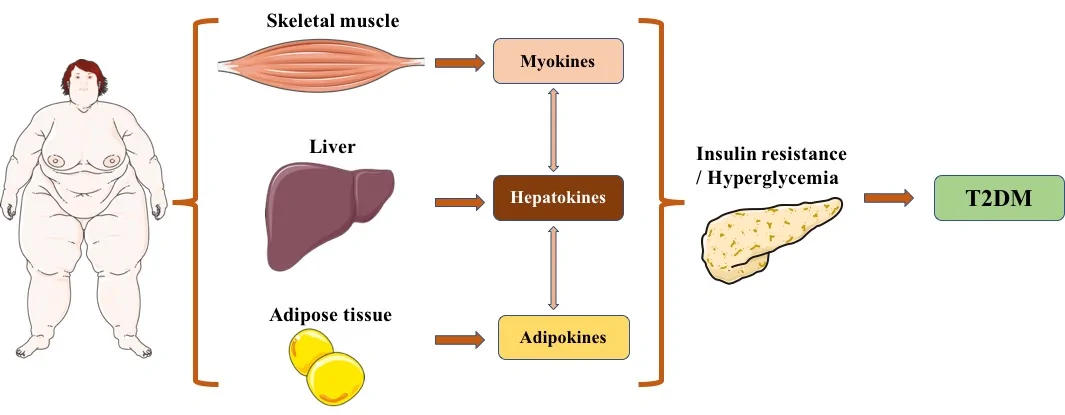
The network of organokines in obesity-related T2DM.

## 3. Crosstalk and Interactions

Obesity is a chronic low-grade inflammatory response state closely associated with an increased risk of metabolic diseases. It is accompanied by alterations in the organokines in the body, such as adipokines, hepatokines, and myokines. Meanwhile, the changes and crosstalk of these organokines further regulate the intricate occurrence and progression of obesity-related metabolic diseases. Adipose tissue plays a crucial role in regulating metabolic balance, not only participating in energy storage, but also acting as a key endocrine organ, secreting adipokines with pro-inflammatory or anti-inflammatory properties, and participating in obesity-related glucose and lipid metabolism. Hepatokines secreted and synthesized by skeletal muscle can play a role in autocrine, endocrine, and paracrine ways to modulate the energy, glucose and lipid metabolism of the organism, which is closely related to obesity, diabetes, and other metabolic diseases. Myokines not only act on skeletal muscles themselves, regulating their metabolism of glucose, lipids and proteins, but also reach the periphery through the blood circulation, serving as messengers between skeletal muscle, fat tissue, and liver. Obesity occurs when energy expenditure is less than energy intake and is featured by accumulation of adipose tissue. Adipose tissue releases free fatty acids, leading to ectopic lipid deposition in the skeletal muscle and liver, which eventually contributes to the progression of metabolic diseases. There is increasing evidence that obesity is closely associated with the development of T2DM. Recent studies have shown that adipokines, hepatokines and myokines play a crucial role in obesity-related T2DM (Fig. 2).

It is well known that T2DM is caused by insufficient insulin secretion or insulin resistance. Moreover, the oxidative stress and chronic inflammation play a crucial role in insulin resistance, thus affecting the progression of T2DM. Organokines can be divided into pro-inflammatory factors and anti-inflammatory factors, which exert different functions or modulate several signaling pathways in T2DM by playing corresponding roles in interorgan crosstalk. In this section, we will particularly emphasize the crosstalk role of the metabolites from adipose tissue, liver, and muscles in T2DM, since these organokines play an important role in insulin biology, as well as in glucose and lipid metabolism.

## 4. Pathophysiological Impact on T2DM

Leptin modulates body mass and food intake by affecting satiety and appetite and is involved in proinflammatory immune responses and lipolysis. As the name suggests, leptin resistance is manifested as an abnormal increase in leptin levels in the blood, but the body is not sensitive to leptin, which prevents it from exerting its corresponding beneficial functions and ultimately leads to obesity. The expression of reduced LEPR, the disruption of signaling pathway and the inability of leptin to reach the target cells may all lead to leptin resistance [96]. Recent studies have revealed that knocking out the LEPR or leptin coding gene in mice leads to insulin resistance, hyperphagia, and obesity [97, 98]. Studies have shown that elevated leptin concentrations are associated with insulin resistance and the progression of T2DM [99]. Moreover, high leptin levels are involved in increased micro-vascular and macro-vascular diabetic complications [100]. The signaling pathway of leptin is modulated by diverse hormones, neurons, and metabolic signals. It has been demonstrated that leptin supplementation can improve insulin resistance and thus play a therapeutic role in T2DM [101]. The level of circulating leptin is directly proportional to adipose mass. Obese subjects experience hyper-leptinemia, but long-term high levels of leptin can lead to leptin resistance. In order to counteract the impact of leptin resistance, leptin levels further increase, unfortunately leading to more severe leptin resistance and forming a vicious cycle. Therefore, recent research has highlighted therapeutic approaches that improve leptin sensitivity in T2DM. Besides, studies have shown that the level of circulating adiponectin is decreased in obesity and T2DM [102, 103]. Reduced adiponectin concentration can increase the risk of obesity-related T2DM, which may be related to the following pathophysiology functions [104-106]. First of all, adiponectin improves insulin resistance by moderating inflammation and oxidative stress. Secondly, adiponectin can lower blood glucose through improving the utilization of fatty acids and glucose in skeletal muscle, as well as preventing glycogenolysis and gluconeogenesis in liver. Thirdly, it has been demonstrated that increased concentration of adiponectin reverses β-cell damage and impaired insulin secretion in obesity-related T2DM. Studies have found that exercise, anti-diabetic drugs, and hypolipidemia drugs can up-regulate the level of adiponectin. Moreover, the level of resistin is elevated in obesity and the patient with T2DM. In obese and hyperglycemic mice, the administration of anti-resistin antibody reduced glucose level and improved insulin sensitivity, indirectly suggesting that elevated resistin levels played a causative role in the high risk of obesity-related T2DM [107]. In addition, it has been indicated that the CTRPs promote the initiation and progression of obesity-related metabolic disorders by directly or indirectly regulating a variety of target proteins involved in insulin signaling, inflammatory pathways, and energy metabolism [108]. For example, Pan et al. confirmed that CTRP1 concentrations were positive correlated with insulin secretion as well as sensitivity inT2DM [109].

Hepatokines also play a positive role in homeostasis, but the dysregulation and imbalance of hepatokines may cause the occurrence of T2DM. It has been reported that SHBG is inversely related to insulin resistance and glucose levels in T2DM [110]. Tibblin et al. found that even after adjusting for underlying factors, low SHBG concentrations were markedly connected with an increased risk of T2DM [111]. Besides, Misu et al. reported that SEPP mRNA expression was up-regulated in liver samples from T2DM patients [112]. They further demonstrated that administration of SEPP complicated insulin resistance both in myocytes and hepatocytes. Yang et al. also confirmed that high levels of SEPP could cause insulin resistance in both in vitro and in vivo experimental [113]. Moreover, the expression of SEPP level was opposite to that of adiponectin, and the increase of SEPP was related to the decrease of adiponectin in patients with T2DM [114].

In addition, myokines play a crucial role in T2DM. Studies have found that the level of irisin in T2DM patients with chronic complications was lower than in those who without chronic complications [115]. Chung et al. confirmed that the level of serum myostatin was positively corelated with diabetic retinopathy [116]. The complex role of BDNF in T2DM involves platelet reactivity, crosstalk with other organokines, and the modulation of various neurotransmitters [117].

## 5. Therapeutic Potential

Obesity-related T2DM has gradually become a major cause of high mortality and morbidity worldwide. Therefore, it is necessary to study and explore different biomarkers and treatments for obesity-related T2DM. Organokines including adipokines, hepatokines and myokines are undoubtedly involved in the development, diagnosis, and potential treatment of obesity-related T2DM. Given the increasing interest in obesity-related T2DM, they have emerged as new clinical application for obesity-related T2DM. In terms of clinical treatment, organokines including adipokines, hepatokines and myokines have the potential to become therapeutic targets for obesity-related T2DM. Nevertheless, it is important to consider the various unpredictable side effects. Therefore, applying organokines to the clinic is a challenging but interesting endeavor that requires further exploration. In the future, targeted delivery systems or drugs designed to be highly specific to organokines receptors will be used to treat obesity-related T2DM.

## 6. Conclusions and Future Directions

Obesity has become a prominent health problem globally and is closely associated with many chronic diseases such as T2DM, cardiovascular diseases, and so on. T2DM is a major metabolic and aging related disease. Which affects millions of people around the world, significantly reducing the quality of life, and causing serious consequences for our health care system. The biggest harm of diabetes is vascular related complications, which lead to amputation, renal failure, stroke, and bring heavy burden to the country and individuals. Recent data suggests that organokines, such as adipokines, hepatokines and myokines, play an important role in tissue crosstalk during the development of T2DM. Obesity-related T2DM becomes a heavy burden of society. Massive evidence proves that organokines including adipokines, hepatokines and myokines are undoubtedly involved in the development, diagnosis, and potential treatment of obesity-related T2DM. Therefore, it is necessary to further understand the roles of organokines in obesity-related T2DM. However, its clinical application still faces some challenges. Systems biology provides an innovative strategy for studying interactive processes from a holistic perspective and has the potential to become a powerful tool for elucidating their underlying mechanisms and developing effective therapeutic tools. In-depth understanding of the mechanisms and networks of adipokines, hepatokines and myokines from different perspectives will provide new insights into the prevention, diagnosis and therapy of obesity-related T2DM.
